# Genetic Alterations in Gastric Cancer Associated with *Helicobacter pylori* Infection

**DOI:** 10.3389/fmed.2017.00047

**Published:** 2017-05-02

**Authors:** Claudia I. Rivas-Ortiz, Yolanda Lopez-Vidal, Luis Jose Rene Arredondo-Hernandez, Gonzalo Castillo-Rojas

**Affiliations:** ^1^Programa de Inmunología Molecular Microbiana, Departamento de Microbiología y Parasitología, Facultad de Medicina, Universidad Nacional Autónoma de México (UNAM), Mexico City, Mexico; ^2^Programa de Microbioma, Facultad de Medicina, Universidad Nacional Autónoma de México (UNAM), Mexico City, Mexico

**Keywords:** gastric cancer, polymorphisms, desoxyribonucleic acid methylation, inflammation, deregulation and overexpression/gene inactivation

## Abstract

Gastric cancer is a world health problem and depicts the fourth leading mortality cause from malignancy in Mexico. Causation of gastric cancer is not only due to the combined effects of environmental factors and genetic variants. Recent molecular studies have transgressed a number of genes involved in gastric carcinogenesis. The aim of this review is to understand the recent basics of gene expression in the development of the process of gastric carcinogenesis. Genetic variants, polymorphisms, desoxyribonucleic acid methylation, and genes involved in mediating inflammation have been associated with the development of gastric carcinogenesis. Recently, these genes (interleukin 10, Il-17, mucin 1, β-catenin, CDX1, SMAD4, SERPINE1, hypoxia-inducible factor 1 subunit alpha, GSK3β, CDH17, matrix metalloproteinase 7, RUNX3, RASSF1A, TFF1, HAI-2, and COX-2) have been studied in association with oncogenic activation or inactivation of tumor suppressor genes. All these mechanisms have been investigated to elucidate the process of gastric carcinogenesis, as well as their potential use as biomarkers and/or molecular targets to treatment of disease.

## Introduction

Gastric cancer is a health problem, because it is the sixth leading cause of death worldwide (1). In Mexico, gastric cancer is the fourth leading cause of death from malignancy in men and women (2). Among the risk factors associated with the development of gastric cancer are smoking, obesity, and consumption of foods high in nitrates (smoked foods and salt, for example). However, *Helicobacter pylori* (*H. pylori*) infection is the main risk factor in the development of gastric cancer, a Gram-negative bacillus that has a spiral morphology when it colonizes the gastric mucosa of human stomach. This bacillus is recognized their participation in the development of atrophic gastritis, peptic ulcer (gastric and duodenal ulcer), and intestinal type gastric cancer. World Health Organization through the International Agency for Research on Cancer recognized *H. pylori* as a carcinogen category I in humans, in 1994; later, in 2009, this was ratified by the collaborative group in cancer and *Helicobacter* (3).

Most gastric cancer patients are asymptomatic and may have advanced disease at diagnosis. These patients may receive potentially curative resection (total or subtotal gastrectomy and lymphadenectomy); therefore, the overall rate at 5 years of patient survival is ranges from 10 to 30% (4). Early gastric cancer is surgically curable, but only discovered during programs of screening for gastric cancer, that unfortunately not widely performed in the world, except for countries, such as Japan, China, Venezuela, and Chile that have a very high incidence of gastric cancer (5–7).

Molecular studies prove that gastric cancer emerges from the combination of environmental factors and a host genetic component. It is noteworthy that the accumulation of genetic and epigenetic alterations plays an important role in the process of carcinogenesis. This review focuses on recent aspects of gene expression in the development of gastric carcinogenesis.

## Host Genetic Susceptibility

Molecular genetic studies have described some variants that are useful as biomarkers of genetic susceptibility involved in the development of gastric cancer. Single-nucleotide polymorphisms (SNPs) are a variation in desoxyribonucleic acid (DNA) sequence that affect a single base [adenine (A), thymine (T), cytosine (C), or guanine (G)] of a genome sequence. SNPs are considered a form of point mutation that has been evolutionarily successful enough to notice a significant part of the population of a species. Genetic variations may modulate the effects of exposure to environmental factors by regulating biological pathways during gastric carcinogenesis.

Genetic variants in genes from cytokines and their receptors associated with inflammation are considered to participate to tumor initiation and promotion. Considering genetic polymorphisms in gastric cancer, this approach has led to a growing interest in recent years. Recently, in the meta-analysis performed by Zhuang et al., found that a promoter polymorphism, in the interleukin 10 (-592C>), might be associated in Asians with gastric cancer development (8). Polymorphisms in the promoter of the gene encoding the tumor necrosis factor (TNFα-308) and interleukin 10 (IL-10) (IL-10-592 and -1082) also have an important role in the development of gastritis associated by *H. pylori* infection. They have been found to modify the risk for the development of gastric cancer in Caucasians (9–12). Wu et al. found an association between IL-17F A7488G and intestinal type gastric cancer (13). This is interesting because the polymorphism of IL-17F 7488 is associated with an increased inflammation by *H. pylori* infection (14). In a meta-analysis of a group of polymorphisms in inflammation-related genes, genes encoding proteins of interleukins [IL-1β, interleukin antagonist receptor, interleukin 8 (IL-8), and IL-10] and TNF-α were investigated. The results show that interleukin antagonist receptor 2 polymorphism represents an increased risk of intestinal cancer in non-Asian populations (15).

Among the genetic variants related to inflammation and its association with gastric cancer caused by *H. pylori* infection. It has been proposed that persistent chronic gastritis during *H. pylori* infection generates a microenvironment where inflammatory mediators activate signaling pathways that may lead to gastric tumorigenesis. The chronic infection of the stomach with *H. pylori* is associated with mutations in genes of epithelial cells, inhibition of apoptosis, stimulation of angiogenesis, and increased cell proliferation. Epithelial cells of the mucosa, including the gastric epithelium, express several cell surface receptors, which indicate the presence of mucosal pathogens and activate inflammatory pathways (16). Mucin (MUC), glycosylated proteins that play important roles protecting epithelial cells and pathogens, are involved in the process of epithelial renewal and differentiation. MUC6 and MUC1 are secreted in the stomach and can play an important role in the development of gastric cancer (17). Recently, studies of the genome-wide association in Japanese and Chinese populations identified chromosome 1q22 harboring the gene MUC1 as a locus that confers susceptibility to the development of gastric cancer. This gene encodes MUC bound to the cell membrane. MUC1 is known as an oncogene with anti-apoptotic function in cancer cells; however, in normal gastric mucosa, it is believed that this protein has a role in protecting gastric epithelial cells from attacks that cause inflammation and carcinogenesis (18).

In its role against cancer, MUC1 consists of two subunits: N- and C-terminal. MUC1-C has a transmembrane domain and a cytoplasmic tail that contains several phosphorylation sites and a binding site to β-catenin. Threonine phosphorylation is contained in the cytoplasmic tail interactions with MUC1 and promotes β-catenin, leading to gene regulation of *p53* (19). It is proposed that the virulence factor cytotoxin-associated protein (CagA) destabilizes the β-catenin/E-cadherin complex located in the cytoplasm of epithelial cells and increases the accumulation of β-catenin in the nucleus. CagA activates β-catenin-dependent genes involved in intestinal differentiation, such as, caudal type homeobox 1 (*CDX1*) transcription factor expressed in the intestine, where it regulates of cell proliferation and differentiation. In areas of the stomach and esophagus with intestinal metaplasia aberrant expression of CDX1 has been found. Therefore, CDXI-induced CagA may play a role in the transdifferentiation of gastric mucosa to intestinal type. Indeed, induction of CDX1 by CagA–β-catenin complex is associated with ectopic expression of intestinal differentiation marker MUC2 in gastric epithelial cells. These genes CDX1 and MUC2 favor the development of intestinal metaplasia, which is a pre-neoplastic lesion (20). Also, the activation of the expression of IL-8, an inflammatory chemotactic cytokine, is induced by the nuclear accumulation of β-catenin. It has been shown that *H. pylori* regulate the increased expression of MUC1 in gastric cancer cells by CpG hypomethylation and signal transducer and activator of transcription 3. This cascade can exist in normal gastric epithelium as an anti-cancer mechanism against infection by *H. pylori* (21).

One of the most important signaling pathways of tumor suppressor genes is transforming growth factor beta (TGF-β), which plays a critical role in regulating cell growth and cell differentiation. Generally, signaling is started with the ligand-induced oligomerization of serine/threonine receptor kinases and the phosphorylation of cytoplasmic signaling molecule Smad. At present, the loss of the nuclear expression of SMAD4 has been described in the progression of gastric cancer (22, 23). Because of the function SMAD4 has in tumor suppression in gastric cancer, Wu et al. conducted a study of five SNPs, finding an association between the C allele (rs177663887) and G allele (rs12456284) with an increased expression of SMAD4 and low risk of gastric cancer (24).

The serpin peptidase inhibitor, clade E, member 1 (SERPINE1) plays a key role in tumorigenesis. It prevents the excessive proteolysis of the extracellular matrix, which is necessary to capillary morphogenesis, cell migration, and invasion. A study by Ju et al. found that a SERPINE1 polymorphism (c.1162+162C>T) in intron 7 is strongly associated with the susceptibility to diffuse gastric cancer (25). Many articles talk about the partnership that exists between genetic polymorphisms and the risk of developing gastric cancer; host genetic factors are very important to the development of cancer. The interaction of different polymorphisms with environmental factors is an effective formula that triggers cancer development and provides us with information to explain the various mechanisms of carcinogenesis.

## Molecular Alterations in Gastric Cancer

Understanding which molecular mechanisms and alterations are carried out after the initiation and progression of gastric tumorigenesis is extremely important for the early detection of the disease and the identification of some therapeutic targets as well. We have identified certain genetic and epigenetic molecular abnormalities in gastric cancer that can help us to decipher the mechanisms of gastric carcinogenesis.

## Genetic Abnormalities

The aberrant cellular metabolism is an important feature during tumorigenesis and cancer progression (26, 27). Programming the energy metabolism is an emerging hallmark of cancer (28); cancer cells reprogram their metabolism by increasing glycolysis instead of mitochondrial oxidative phosphorylation to generate cellular energy (29). Tissue hypoxia is a fundamental driving force that leads to the reprogramming of cell metabolism (30). In stomach cancer, a low percentage of cells are in the G0 phase of the cell cycle, producing accelerated cell proliferation. When the level of hypoxia increases, the activity level of oxidative phosphorylation as the main energy (ATP) producer decreases and glycolysis increases, allowing cancer cells to accelerate the uptake of glucose from the bloodstream to compensate for the loss of energy production, which is necessary for the cells to remain viable. This change in energy metabolisms accelerated cell proliferation and increased hypoxia, generating a vicious cycle of enhanced growth of cancer. This cycle is broken down when the angiogenesis occurs, and a high level of hypoxia triggers it. When this happens, there is a reduction in hypoxia levels and switches back to oxidative phosphorylation as the main energy producer. This process continues until the cells return to hypoxic and the levels of cellular hypoxia rise and fall periodically, which agrees with the increase in cancer mass and new angiogenesis, respectively. Under an atmosphere of hypoxia increased cell proliferation and cancer progression (31).

At a gene level, hypoxia-inducible factor 1 (*HIF-1*) is the primary transcriptional activator sensitive to oxygen that helps cells to adapt to low oxygen tension (hypoxia) (32). *HIF-1* consists of a constitutively expressed β-subunit and a hypoxia-inducible α-subunit. The latter (*HIF-1α*) only stabilized in hypoxic conditions and regulated the transcriptional activity of *HIF-1* (33). Overexpression of *HIF-1α* is involved in the activation of multiple target genes crucial in cancer biology, including erythropoiesis, angiogenesis, glucose metabolism, cell proliferation, survival, and apoptosis (34). It has been shown that the expression of over 20 genes is directly regulated by *HIF-1α*; among these genes is *NFκB1*, a regulatory molecule of inflammation and cancer (35).

Zheng et al. reported overexpression of the inactive form of glycogen synthase kinase 3 beta (GSK3β) and phospo-GSK3β (ser9) in gastric cancer compared to normal mucosa. This article addresses that overexpression of phospo-GSK3β (ser9) is positively correlated with prognosis of gastric cancer (36); phospo-GSK3β (ser9) is induced by gastrin and leads to GSK3β inhibition, increased Snail expression, nuclear translocation of β-catenin, and an increase in cell migration in gastric cancer (37).

Transmembrane proteins are proteins involved in signal communication between the extracellular and intracellular spaces. It has been described that the transmembrane protein in gastric cancer CD133 is overexpressed in 57% of gastric cancer and positively correlates with the expression of Ki-67 (38). A member of the cadherin superfamily, CDH17, was reported to be a marker for gastric cancer in early stage. CDH17 expression was positively associated with a good prognosis of gastric cancer (39). Another component of the extracellular matrix is hyaluronic acid (HA). The two known cellular receptors of HA are cluster of differentiation 168 (CD168) and cluster of differentiation 44 (CD44). The overexpression of CD168 was reported in a panel of gastric cancer cases, where CD168 expression was associated with the depth of invasion and cancer metastasis (40). In another study, Xie et al. described that tumor stem cell (SC) surface marker CD44 (CD44v6) is involved in gastric cancer metastasis. They observed the overexpression of CD44v6 in all cancer cell lines. The 5-year survival rate of patients with CD44v6 positive expression is significantly worse compared to the survival rate of patients with CD44v6 negative expression (41). Wang et al. performed a meta-analysis to quantitatively study the correlation of CD44 expression with the clinicopathological data of patients with gastric cancer. They found that CD44 expression was related to stage, tumor size, and lymph node metastasis of gastric cancer, whereas CD44v6 was related to lymph node metastasis, lymphatic invasion, and venous invasion (42).

Matrix metalloproteinases (MMPs) are enzymes that generate proteolysis, are zinc dependent, and are involved in diverse physiological and pathological processes, including degradation of extracellular matrix, tissue remodeling, inflammation, tumor invasion, and tumor cell metastasis. Due to the functioning of MMP their operation in cancer, Wang and colleagues performed a meta-analysis that showed the expression levels of MMP-7 were increased in gastric cancer. They associated it with lymph node metastasis, advanced tumor node metastasis (TNM) stage, and invasion of the tumor. Therefore, MMP-7 can be a useful biomarker for determining the progression of gastric cancer and prognosis (43). The increased expression of MMP-9, -12, and -21, is associated with decreased survival in gastric cancer (44).

Epithelial–mesenchymal transition (EMT) plays a significant role in tumor progression and invasion. *Snail* is a known regulator of EMT in various malignancies. After the analysis of gene expression and complementary DNA microarray, it was observed that the overexpression of *Snail* increases invasion, cell migration, and tumor progression in gastric cancer (45). This gene can also be used as a predictive biomarker for prognosis or aggressiveness in gastric cancer.

It is unclear whether *H. pylori* promote gastric cancer by direct invasion of the epithelium and interaction with oncoproteins. Although bacteria are not invasive and hide in the mucus layer to prevent stomach acid, we observed that about 10% of them adhere to the cells, and some intracellularly, but the cancer process is still unknown. There is evidence implicating the CagA as a promoting oncoprotein in human gastric cancer. CagA is a 120–145 kDa protein encoded by the cytotoxin-associated gene A (*cagA*) gene, located in the *cag* pathogenicity island (*cag*-PAI); it also encodes the type IV secretion system with which CagA is injected into the epithelial cells of the gastric guest (46). Infection with strains possessing this protein is associated with a greater degree of inflammation in gastric mucosa; in severe gastric atrophy, it is considered an important process in the development of gastric cancer (47–49).

CagA translocation to epithelial cells allows the interaction with host proteins in two different forms: phosphorylation-dependent and independent from each other. CagA is phosphorylated by the Src kinase (SFK) family and thus activates the tyrosine phosphatase (SHP-2) (50), altering cell adhesion and migration (51) and stimulating cell-signaling pathway Erk (52). CagA phosphorylated by SFK can be dependent and independent from Ras (53), whereby uncontrolled cell proliferation is promoted (49, 54). It also produces an aberrant epithelial cell adhesion and cytoskeletal rearrangement (55). Moreover, unphosphorylated CagA can deregulate different signaling cascades in epithelial cells, resulting in alterations that cause cell proliferation and the mobility of the cells with increased expression of genes involved in oncogenic transformation (56–58). CagA mediates the enhanced surface expression of epidermal growth factor receptor (EGFR) by inhibiting receptor endocytosis. The EGFR controls crucial cellular processes such as cell division or cell death as well as motility and matrix adhesion. Thus, a deregulation of the EGFR caused by increased receptor surface expression or mutations in the extracellular domain is linked to cancer development. In addition to its involvement in carcinogenesis, EGF signaling has been implicated in the inhibition of gastric acid secretion and liked to impaired wound healing of the gastric epithelial layer, two factors involved in the development of gastric ulcer (59).

Constitutive EGFR activity reduces acid secretion but also predisposes oncogenic transformation, supporting signaling deregulation as a direct cause of carcinogenesis. Furthermore, the association of CagA with protease active receptor (PAR1), a member of polarity complex, appears to disturb the epithelial cell polarity, promoting interaction between CagA and tyrosine phosphatase SHP-2, which activates extracellular regulated kinases by signals (ERK1/2) and promotes EMT (60). For all this, it seems that CagA houses adapter proteins and kidnaps signaling pathways to maintain a non-polarized phenotype with attenuated gastric acid secretion, which is favorable for bacterial survival (Table 1).

**Table 1:** **Genes activated in the presence of CagA**.

CagA	Gene	Function	Reference
Unphosphorylated	*E-cadherin*	Cell adhesion	(20)
	*β-catenin*	Adherens junctions	(20, 21)
	*c-Met*	Cell proliferation	(56)
	*PLC-γ*	Cell migration	(56)
	*Grb2*	Cell proliferation and mobility	(57)
	*Par1*	Cell polarity	(60)
	*EGF*	Inhibitor of acid secretion	(59)
	*EGFR*	Inhibitor of acid secretion	(59)
Phosphorylated	*SHP-2*	Cell migration	(50, 51)
	*Erk*	Cell proliferation	(48, 52–54)
	*MAP kinase*	Signal transduction	(61)
	*FAK kinase*	Cell motility	(51, 62)

**Figure 1:**
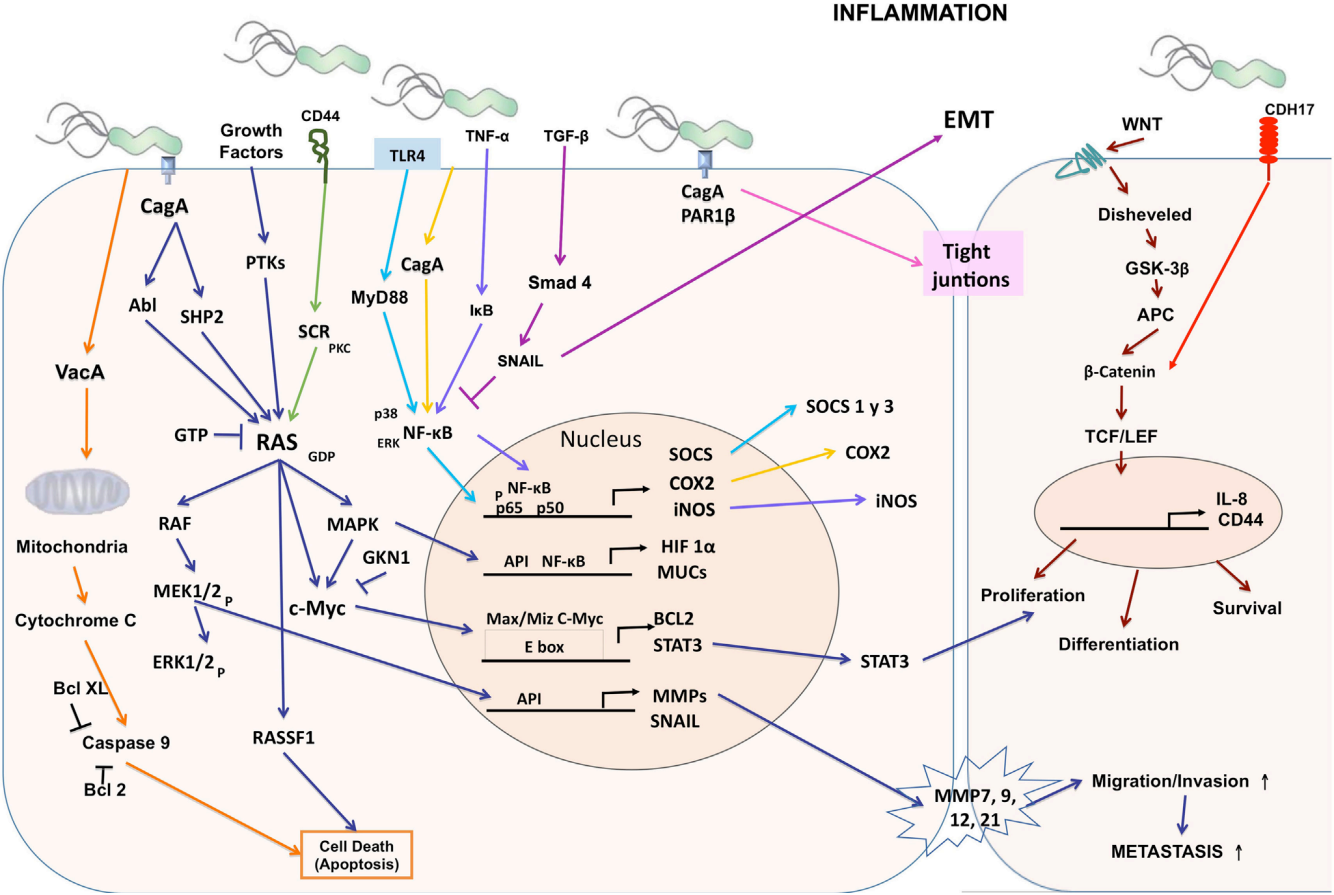
**Cellular and molecular pathogenesis of *Helicobacter pylori* infection in gastric carcinogenesis**. The phosphorylated CagA active in the SHP-2/MAPK pathway regulates MEK/extracellular signal-regulated kinase (ERK), RAS/cMyc, and NF-κB pathways as a result of the regulation of genes such as hypoxia-inducible factor 1 subunit alpha (HIF-1α), mucins (MUCs), suppressor of cytokine signaling (SOCS), COX-2, inducible nitric oxide synthase (iNOS), BCL2, signal transducer and activator of transcription 3 (STAT3), matrix metalloproteinases (MMPs), and SNAIL producing proliferation, differentiation, cell survival, and increased migration, invasion, and metastasis of cancer cells. CagA alters the tight junctions independently of phosphorylation. Vacuolating cytotoxin (VacA) alters the permeability of the mitochondrial membrane and favors apoptosis.

The Runt-related transcription factor 3 (RUNX3) gene is a tumor suppressor in many tissues and often inactive in gastric cancer. Tsang et al. showed that the virulence factor of *H. pylori* CagA is capable of binding RUNX3, inducing ubiquitination and degradation of RUNX3 by proteasome machinery (63). This tumor suppressor plays an important role in immune regulation, while its reduced expression is related to the development and progression of gastric carcinoma. The levels of expression of *RUNX3, T-bet*, and *IFN-*γ are lower in patients with gastric carcinoma than in control groups and may contribute to the development of cancer (64) (Figure 1).

## Epigenetic Changes Promote the Development of Gastric Cancer

Desoxyribonucleic acid methylation is an important epigenetic modification that is involved in the control of gene expression. It is a covalent chemical modification catalyzed by the DNA methyltransferase family of enzymes involved in the addition of a methyl group (CH_3_) on the fifth carbon of cytosine (65). DNA methylation is grouped in regions known as CpG islands, which are regions of DNA localized in up to 40% of promoters of genes. The methylation within a gene promoter is associated with the transcriptional inhibition of gene expression, particularly in tumor suppressor genes.

There is information regarding the roles of many tumor suppressor genes in cancer prevention; among those roles are DNA repair, cell adhesion, cell cycle control, and apoptosis. However, these genes are silenced by hypermethylation of the promoters during carcinogenesis. Numerous studies on DNA methylation report the existence of more than 100 tumor suppressor genes in gastric cancer, including *E-cadherin, RASSF1A, p16, GSTP1, SOCS1, SFRP1*, and *PTEN* (66).

E-cadherin is one of the most important suppressor genes in gastric cancer tumors. Inactivation of E-cadherin increases proliferation, invasion, and metastasis, which contribute to tumor progression. It is known that silencing of E-cadherin is due to CpG island methylation in undifferentiated-type gastric cancer (66). *RASSF1A* is part of the Ras association domain family, another group of tumor suppressor gene that plays a critical role in cell cycle regulation, apoptosis, and microtubule stability, by regulating signaling pathway Ras (67). Also, *RASSF1A* methylation is closely associated with advanced cancer, according to the TNM (68) and poor prognosis in patients with gastric cancer. Thus, it represents an objective diagnosis and a potential therapy for gastric cancer. *H. pylori* infection contributes to the loss of expression of the transcription factor related Runt-3 (RUNX3) in gastric cancer through its overmethylation (69).

Nakajima et al. demonstrated that methylation levels occurring in the gastric mucosa are increased in patients with *H. pylori* positive gastric cancer (70). The inflammation caused by infection with *H. pylori* is essential for DNA methylation of promoters of genes related to inflammation, as *IL-1β, NOS2*, and *TNF* (71, 72). Methylation induced by *H. pylori* infection in the promoter of E-cadherin, the component involved in maintaining stability of the epithelial extracellular matrix, is mediated by *IL-1β* (73). The eradication of *H. pylori* infection reduces the methylation of E-cadherin promoter, demonstrating that infection with *H. pylori* is strongly related to DNA methylation in gastric cancer (74). DNA methylation induced by *H. pylori* in precancerous lesions might be a new approach for the prevention of gastric cancer.

Zong et al. performed a genetic-level analysis of DNA methylation of gastric cancer cell lines, gastric cancer samples obtained from gastrectomies, normal gastric mucosa, and non-cancerous gastric mucosa where they confirmed that the *OSR2, PPFIA3*, and *VAV3* genes are poorly methylated (5% or less) in non-cancerous and highly methylated (85% or more) cells in cancer cells. These three genes may be useful for estimating the fraction of cancer cells in gastric cancer (75).

Recent comprehensive analyses showed that many cancer-related pathways are further altered by aberrant DNA methylation than by mutations. It is known that in the non-cancerous gastric mucosa, DNA methylation occurs, which produces an epigenetic environment for the development of carcinogenesis. Also, the chronic inflammation induced by *H. pylori* infection induces aberrant DNA methylation. Expression of the *IL-1β, NOS2*, and *TNF* genes are highly associated with the induction of aberrant DNA methylation. The degree of aberrant DNA methylation is correlated with the risk of gastric cancer. It is known that the induction of DNA methylation is the main route by which *H. pylori* infection induces gastric cancer; this information may be useful to decrease gastric cancer (76).

Gene silencing in gastric cancer may occur mainly by point mutations, loss of heterozygosity, and promoter hypermethylation. A tumor suppressor gene whose expression is frequently deregulated in gastric cancer by hypermethylation of the Trefoil factor 1 gene (*TFF1*) whose function is to defend and repair of gastrointestinal mucosa. All normal gastric tissues express *TFF1*, but Im and co-workers have been shown to reduce the expression of this gene in gastric cancer (77). A recent study reported that polypeptide hormone gastrin is secreted by the glands of the pyloric antrum of the stomach, stimulating the secretion of hydrochloric acid and pepsinogen. It is worth noting that gastrin suppresses the *TFF1* promoter (78).

The pancreatic duodenal homeobox transcription factor 1 (*PDX1*) is an important gene in cell differentiation and development of the pancreas, duodenum, and antrum. It is a tumor suppressor gene with an expression that is frequently deregulated in gastric cancer. *PDX1* is often expressed in normal gastric glands but is absent in human gastric cancer cell lines and gastric cancer. *PDX1* silencing is given by promoter hypermethylation and histone hypoacetylation (79, 80). In gastric cancer in several tumor suppressor, genes and tumor-related genes are silenced by promoter methylation of gene, generating loss of protein expression. Mikata et al. showed that BCL2L10, which belongs to the family of pro-apoptotic Bcl-2, is silenced by hypermethylation of the promoter. This study suggests that silencing BCL2L10 by methylation is a common feature in gastric cancer and may be involved in the early stages of gastric carcinogenesis (81).

In humans, X-ray repair cross-complementing protein 1 (XRCC1) is encoded by the XRCC1 gene involved in DNA repair due to promoter hypermethylation; the expression of this gene is regulated in gastric carcinogenesis (82). Serine protease inhibitor kunitz type 2 (SPINT2/HAI-2) has been implicated in tumor pathogenesis through its influence on cell cycle progression, tumor cell migration and invasion, angiogenesis, and protection from apoptotic stimuli. Promoter methylation and transcriptional silencing of HAI-2/SPINT2 is significantly linked to cell differentiation and metastasis in gastric cancer (83, 84).

Glutamate receptor ionotropic kainate 2 (GRIK2) is a protein that plays a tumor suppressor role in gastric cancer. GRIK2 is often highly methylated in cell lines and gastric cancer primary tumors, but not in normal adjacent tissues, leading to gene silencing (85). To date, many genes with different biological functions are found methylated in gastric cancer (Table 2).

**Table 2:** **Prevalence of gene methylation in gastric cancer**.

Function	Gene	Assay	Prevalence of methylation (%)		Reference
			Normal	Cancer	
Cell cycle	*P16*	MSP	3.8–35.0	21.3–45.0	(86)
	*PTEN*	MPS		76	(87)
Adhesion/invasion/cell migration	*E-cadherin/CDH1*	MSP	16.0–36.1	50.6–84.0	(88)
	*GRIK2*	Q-MSP	7.41–30.0	50.0–66.6	(85)
Growth/cell differentiation	*HAI-2/SPINT2*	MSP	0.0	75.0	(83)
Apoptosis	*BCL2*	MethyLight	25.9	80	(89)
	*GSTP1*	MSP	1.9	20.6	(90)
Desoxyribonucleic acid (DNA) repair	*XRCC1*	MPS			(91)
Transcriptional regulation	*RUNX3*	Q-MSP	7.4	56.0–75.2	(92)
	*PDX1*	MSP	16.7	77	(80)
Proliferation	*TFF1*	MPS	-	40.9	(93)
RAS pathway	*RASSF1A*	MPS	5.7	45.6–61.8	(94)
STAT pathway	*SOCS-1*	MPS	12.0	44.0	(95)
	*SOCS-3*	MPS	13.0	74	(96)
WNT pathway	*SFRP1*	MSP	15.0	44.0	(97)

*MSP, methylation-specific PCR; Q-MSP, quantitative methylation-specific PCR; MethyLight, real-time PCR to measure DNA methylation*.

## Cancer Stem Cells (CSCs) and Gastric Carcinogenesis

In the gastrointestinal tract, there are normal SCs, which possess both self-renewal ability and asymmetric cleavage ability to generate progenitor cells that differentiate into epithelial cells. These SCs in the normal gastric mucosal are present in the proliferative region of the neck/isthmus region and experience a complex bipolar migration of the neck/isthmus region upward, thereby becoming differentiated normal epithelial cells (98).

Cancer stem cells are malignant cells with capacities or characteristics of SCs. There have been many studies of CSCs that demonstrate rapid growth and high metastatic potential. Recent evidence suggests the existence of cancerous SCs in solid tumors. This indicates that under conditions, the local microenvironment can promote the development of gastric cancer. *H. pylori* infection and accompanying chronic inflammatory processes provide the critical initiators that favor cell growth and tissue repair response, leading to gastric carcinogenesis (99).

The discovery of CSCs and their characteristics have favored the understanding of the molecular mechanism of tumorigenesis and tumor development, but above all generating new and effective strategies for the treatment of gastric cancer. Gastric CSCs are found in the base for the onset of gastric cancer. They can be derived from gastric SCs in gastric tissues or mesenchymal SCs from bone marrow. As with other SCs, gastric CSCs express drug resistance genes, which make them refractory to conventional therapies, explaining why cancer therapies are far from curative and why cancer relapses are frequent (100). Specific molecular markers have been studied as CD44 and CD133 for the identification and isolation of gastric CSCs. These CSC markers can help in early diagnosis in the classification of gastric cancer (101).

Maeda et al. reports that the aberrant methylation of the DNA that is induced in the SCs of a gastric gland is permanent because the methylation in the SCs would be conserved and reproduced, which would allow to determine the fraction of cells with methylation. Contrary to the methylation that is generated only in the differentiated cells will disappear when they are replaced with new cells without methylation that are derived from some SC without methylation, of important way this information indicates to us that the methylation induced in the differentiated cells will be a transient component (76).

## Inflammation Induced by *H. pylori* is Important in Gastric Carcinogenesis

Several studies attempt to elucidate the mechanism by which *H. pylori* is able to recruit inflammatory cells. It has been shown that antigens extracted from *H. pylori* have *in vitro* chemotactic activity for monocytes and neutrophils (102). Infected gastric mucosa contains a significant population of macrophages that produce nitric oxide, interleukin 6 (IL-6), IL-1β, TNF-α, and interleukin 12 (IL-12), which help to establish a Th1 response responsible to produce IFN-γ and some interleukin 4 (IL-4) and interleukin 5. Smaller dendritic cells are also present and respond to *H. pylori* infection with the production of IL-6, IL-8, IL-10, and IL-12. They have a higher expression of cluster of differentiation 80, cluster of differentiation 83, cluster of differentiation 86, and human leukocyte antigens, because of stimulation with bacteria (103).

Urease antigen was detected *in vivo* in the lamina propria of gastric biopsies of patients infected with *H. pylori*. This led to examine immunomodulating substances secreted in response to *H. pylori* infection, as increased levels of IL-8 were present in *H. pylori*-associated gastritis (104). It is also known that components such as porin (105) and lipopolysaccharides (LPSs) can stimulate the expression of cytokine in polymorphonuclear cells. IL-8 is a chemokine secreted by several cell types, including monocytes, fibroblasts, endothelial cells, and epithelial cells. Its function is to serve as a potent inflammatory mediator for the attraction and activation of polymorphonuclear cells, particularly neutrophils. Since bacteria make contact only with the surface of the gastric epithelium, secretion of IL-8 is produced by the epithelium because of interaction and regulation of inflammatory and immune processes in response to the bacteria.

*Helicobacter pylori* also activate cyclooxygenase (COX), which is an enzyme that catalyzes proinflammatory key steps in the formation of inflammatory prostaglandins. COX-1 is constitutively expressed, whereas COX-2 is induced by cytokines such as TNF-α, interferon gamma (INF-γ), and IL-1 (106). The expression of COX-2 is increased in the gastric mucosa of individuals infected with *H. pylori* (107, 108). The expression of COX-2 is further increased in premalignant lesions (chronic atrophic gastritis and intestinal metaplasia) and malignant lesions induced by *H. pylori* (adenocarcinoma) (109). COX-2 is a key enzyme in the biosynthesis of prostanoids and is the primary target of non-steroidal anti-inflammatory drugs in neoplastic and inflammatory conditions. COX-2 is involved in the processes leading to tumor progression, including angiogenesis, survival, proliferation, invasion, and immunosuppression (91). For this reason, COX-2 is considered a potential therapeutic target in the prevention and treatment of gastric cancer.

The *H. pylori*-induced inflammatory response leads to the release of mutagenic substances, as metabolites of inducible nitric oxide synthase (iNOS), which promote oncogenesis (108, 110). Nitric oxide generated by iNOS may be converted into reactive nitrogen species that modify DNA and proteins. The superoxide anion radicals generated by neutrophils also induce DNA damage *via* formation of DNA adducts (111).

It has been shown that *H. pylori* can employ multiple mechanisms to degrade or destabilize host responses. Another mechanism used to regulate *H. pylori* decreased immune response is the vacuolating cytotoxin. This cytotoxin interferes with processing and presentation of antigens by antigen-presenting cells. It also inhibits the activation of T cells through interference associated with calcineurin signaling pathway of IL-2 (112).

Most persons infected with *H. pylori* develop specific antibodies found in serum, gastric aspirates, or extracts. High titers of IgG and IgA antibodies to membrane proteins, flagellin, urease, and the LPS of *Helicobacter pylori* adhesion A, were reported in patients infected with *H. pylori* (113). On the other hand, López-Vidal et al. found a progressive accumulation of a specific IgA response against *H. pylori* in patients with gastric cancer, suggesting that it participates in the damage and in the development of gastric cancer (114). Gastritis induced by *H. pylori* is produced by bacterial factors that stimulate epithelial cell, macrophage, and dendritic cell activation and induces the recruitment of T CD4+ and CD8+ cells in the gastric mucosa (115). Some studies have shown that the response of T helper cells to infection with *H. pylori* is polarized, as the T CD4+ cells in the gastric mucosa of infected individuals produce proinflammatory cytokines Th1 such as IL-12, and INF-γ, whereas Th2 regulatory cytokines such as IL-4 are absent in *H. pylori* infection (116, 117).

Amedei et al. suggested that neutrophil-activating protein of *H. pylori* (HP-NAP) contributes to Th1 response in the gastric mucosa of patients infected by *H. pylori*. Regulatory T cells are another important subset of cells producing IL-10 and TGF-β (118). There is an infiltration of CD4+ T cells in the gastric mucosa; therefore, several studies have tried to address the inefficiency of the host response in clearing the infection. They have shown that *H. pylori* infection may reduce T cell response and T cell anergy (115). CD45RO+ T cells of memory, and CD69+ and CD25+ activated cells are increased in the lamina propria of the antrum of infected subjects. This suggests that the presence of regulatory T cells, CD4+, and CD25+ in peripheral blood of individuals infected by *H. pylori* could inhibit the response of CD4+ T cells into the bacterium (115, 119). These findings help to explain the inability of the host response to clear the infection due to the activation of regulatory T cells. An increase of T CD4+, CD25high, and FOXP3+ cells was recently found in the gastric mucosa of infected individuals *H. pylori*. Such cells may reduce mucosal damage mediated by T cells as well as the response of specific T cells, possibly by reducing the activation of CD4+ T cells that produce INF-γ. These responses can be effective to protect against infection by this bacterium (115, 119). The role of CD8+ T cells in the gastric mucosa of *H. pylori*-infected individuals is less clear than that of CD4+ T cells. The favorable participation of CD8+ T cells in the immune response to *H. pylori* infection showed that *H. pylori* colonization is associated with a greater numbers of CD8+ lymphocytes in crypt of epithelium. Their role in the local responses is the production of IFN-γ, with increased expression of major histocompatibility complex class II molecules in adjacent cells (120). Despite these immune mechanisms, *H. pylori* infection is not eliminated because the bacteria have a number of mechanisms are evaded or regulate by host responses. Understanding these multiple mechanisms is a necessary step toward the development of immune strategies to protect from the initial infection and to remove the infections that are already established.

**Figure 2:**
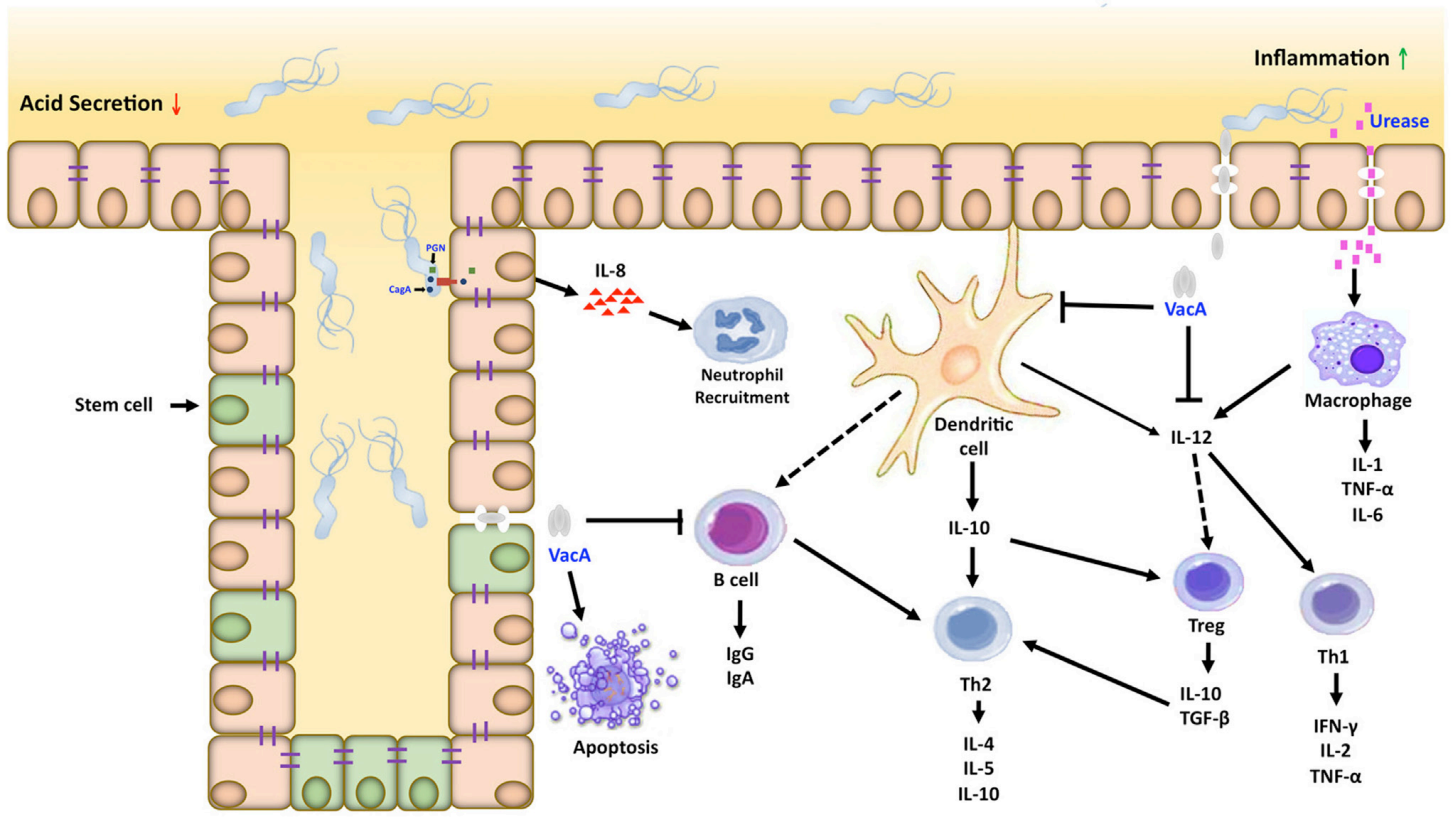
**Inflammatory microenvironment induced by *Helicobacter pylori* (*H. pylori*) in gastric carcinogenesis**. Dendritic cells and macrophages can serve as a bridge between the innate and adaptive immune response directed against *H. pylori* within the gastric mucosa. Interactions between *H. pylori* virulence factors and gastric epithelial cells can activate macrophages and recruit neutrophils, which amplifies the T cell response to this pathogen. Dendritic cells in contact with *H. pylori* antigens activate T cells in different ways, inducing the Th1, Th2/T regulatory response generating proinflammatory and anti-inflammatory cytokines, respectively.

Inflammation is important in the development and progression of gastric cancer; suppressor of cytokine signaling (SOCS) 1 and 3 negatively regulate the signaling of proinflammatory cytokine. We analyzed the expression levels of SOCS-1 and SOCS-3 in gastric tumors and adjacent normal mucosa tissue; the results showed to be lower in the normal mucosa and in the tumor tissues. Also, the low expression of SOCS-1 and SOCS-3 is an indicator of a poor prognosis of gastric cancer (121), which might be of prognostic significance (Figure 2).

## Conclusion

Gastric cancer is a complex disease that emerges from the interaction of multiple processes and factors such as lifestyle, eating habits, and infections, along with genetic variants and molecular alterations acquired during the patient’s life. There are many research works that have been carried out to find molecular markers for gastric cancer; however, there are very few studies that include information about gene expression analysis of the stages of progression of gastric cancer. The real mechanisms are poorly known, and more work is needed to understand the causes of gastric cancer progression in order to obtain biomarkers for early diagnosis, prognosis, and effective treatment of the disease.

## Author Contributions

CIRO, YLV, LJRAH and GCR have substantially contributed to the development of the manuscript outline and literature search strategy, have critically reviewed manuscript drafts for intellectual content, have approved the version to be published, and agreed to be accountable for all aspects of the work in ensuring that questions related to the accuracy or integrity of any part of the work are appropriately investigated and resolved.

## Conflict of Interest Statement

The authors have declared that no competing interests exist, and no relationships or commercial interests with Mexican or foreign companies are related to this review. The reviewer, MS, and handling editor declared their shared affiliation, and the handling editor states that the process nevertheless met the standards of a fair and objective review.

## Abbreviations

DNA, desoxyribonucleic acid; *H. pylori, Helicobacter pylori*; IARC, International Agency for Research on Cancer; SNPs, single-nucleotide polymorphisms; TNF-α, tumor necrosis factor alpha; IL-1β, interleukin 1 beta; IL-1RN, interleukin antagonist receptor; IL-1RN2, interleukin antagonist receptor 2; IL-4, interleukin 4; IL-5, interleukin 5; IL-6, interleukin 6; IL-8, interleukin 8; IL-10, interleukin 10; IL-12, interleukin 12; IL-17F, interleukin 17F; Th, T helper cell; Th1, T helper cell type 1; Th2, T helper cell type 2; IgG, immunoglobulin G; IgA, immunoglobulin A; iNOS, nitric oxide synthases; MUC, mucin; MUC1, mucin 1; MUC1-C, mucin 1 subunit C-terminal; MUC2, mucin 2; MUC6, mucin 6; GWAS, genome-wide association study; β-catenin, beta-catenin; E-cadherin, cadherin epithelial tissue; *cagA*, cytotoxin-associated gene A; CagA, cytotoxin-associated protein; *cag*-PAI, *cag* pathogenicity island; T4SS, type IV secretion system; VacA, vacuolating cytotoxin; LPS, lipopolysaccharide; HpaA, *Helicobacter pylori* adhesion A; HP-NAP, *Helicobacter pylori* neutrophil-activating protein; CDX1, homeobox gene such as flow type 1; CpG, cytosine-phosphate-guanine; STAT3, signal transducer and activator of transcription 3; TGF-β, transforming growth factor beta; SMAD4 or DPC4, SMAD family member no 4 or mothers against decapentaplegic homolog 4, or deleted in pancreatic cancer-4; SERPINE1, serpin peptidase inhibitor clade E, member 1; HIF-1, hypoxia-inducible factor 1; HIF-1α, hypoxia-inducible factor 1 subunit alpha; NF-κB1, nuclear factor kappa-light-chain-enhancer of active B cells class I or p105 subunit; GSK3β, glycogen synthase kinase 3 beta; phospo-GSK3β (ser9), phospho-glycogen synthase kinase 3 beta-antibody (Ser9); Ki-67, marker of proliferation; CDH17, cadherin 17; CD4, cluster of differentiation 4; CD8, cluster of differentiation 8; CD25, cluster of differentiation 25; alpha chain of IL-2 receptor; CD44, cluster of differentiation 44, receptor for hyaluronic acid (HA); CD45RO, protein tyrosine phosphatase receptor type C isoform RO located on memory T cells; CD69, cluster of differentiation 69; CD80, cluster of differentiation 80; CD83, cluster of differentiation 83; CD86, cluster of differentiation 86; CD133, prominin-1; CD168, cluster of differentiation 168; hyaluronan-mediated motility receptor; FOXP3, forkhead box P3 or scurfin; MHC class II, major histocompatibility complex class II molecules; HLA-DR, human leukocyte antigens; GSH, glutathione; MMPs, matrix metalloproteinases; MMP-7, matrix metalloproteinase 7; MMP-9, matrix metalloproteinase 9; MMP-12, matrix metalloproteinase 12; MMP-21, matrix metalloproteinase 21; EMT, epithelial–mesenchymal transition; Snail, zinc finger protein; Src, proto-oncogene tyrosine-protein kinase; SFK, Src family kinase; SHP-2, tyrosine phosphatase 2; ERK, extracellular signal-regulated kinase; Ras, Ras superfamily; EGFR, epidermal growth factor receptor; PAR1, protease-activated receptors; RUNX3, runt-related transcription factor 3; T-bet, T-box transcription factor; IFN-γ, interferon gamma; CH_3_, methyl group; RASSF1A, Ras association domain-containing protein 1; p16, cyclin-dependent kinase inhibitor 2A, multiple tumor suppressor 1; GSTP1, glutathione S-transferase P; SOCS1, suppressor of cytokine signaling 1; SOCS3, suppressor of cytokine signaling 3; SFRP1, secreted frizzled-related protein 1; PTEN, phosphatase and tensin homolog; TNM, tumor node metastasis; NOS2, nitric oxide synthase 2 (inducible); TFF1, trefoil factor 1 gene; PDX1, pancreatic duodenal homeobox transcription factor 1; BCL2L10, Bcl-2-like protein 10; Bcl-2, B-cell lymphoma 2; XRCC1, X-ray repair cross-complementing protein 1; HAI-2 or SPINT2, serine peptidase inhibitor, kunitz type 2; GRIK2, glutamate receptor, ionotropic kainate 2; COX, cyclooxygenase; COX-1, cyclooxygenase-1 or prostaglandin-endoperoxide synthase 1; COX-2, prostaglandin-endoperoxide synthase 2.
